# Hydroxychloroquine decreases human MSC‐derived osteoblast differentiation and mineralization *in vitro*

**DOI:** 10.1111/jcmm.13373

**Published:** 2017-10-03

**Authors:** Tim Both, H. Jeroen van de Peppel, M. Carola Zillikens, Marijke Koedam, Johannes P. T. M. van Leeuwen, P. Martin van Hagen, Paul L. A. van Daele, Bram C. J. van der Eerden

**Affiliations:** ^1^ Department of Internal Medicine Division of Clinical Immunology Erasmus Medical Center Rotterdam The Netherlands; ^2^ Department of Internal Medicine Division of Endocrinology Erasmus Medical Center Rotterdam The Netherlands

**Keywords:** hydroxychloroquine, osteoblast, mineralization, simvastatin, microarray

## Abstract

We recently showed that patients with primary Sjögren Syndrome (pSS) have significantly higher bone mineral density (BMD) compared to healthy controls. The majority of those patients (69%) was using hydroxychloroquine (HCQ), which may have favourable effects on BMD. To study the direct effects of HCQ on human MSC‐derived osteoblast activity. Osteoblasts were cultured from human mesenchymal stromal cells (hMSCs). Cultures were treated with different HCQ doses (control, 1 and 5 µg/ml). Alkaline phosphatase activity and calcium measurements were performed to evaluate osteoblast differentiation and activity, respectively. Detailed microarray analysis was performed in 5 µg/ml HCQ‐treated cells and controls followed by qPCR validation. Additional cultures were performed using the cholesterol synthesis inhibitor simvastatin (SIM) to evaluate a potential mechanism of action. We showed that HCQ inhibits both MSC‐derived osteoblast differentiation and mineralization *in vitro*. Microarray analysis and additional PCR validation revealed a highly significant up‐regulation of the cholesterol biosynthesis, lysosomal and extracellular matrix pathways in the 5 µg/ml HCQ‐treated cells compared to controls. Besides, we demonstrated that 1 µM SIM also decreases MSC‐derived osteoblast differentiation and mineralization compared to controls. It appears that the positive effect of HCQ on BMD cannot be explained by a stimulating effect on the MSC‐derived osteoblast. The discrepancy between high BMD and decreased MSC‐derived osteoblast function due to HCQ treatment might be caused by systemic factors that stimulate bone formation and/or local factors that reduce bone resorption, which is lacking in cell cultures.

**Key messages**

- HCQ treatment leads to decreased MSC‐derived osteoblast differentiation and mineralization *in vitro*
- HCQ significantly up‐regulates the cholesterol metabolism pathway and lysosomal pathway, which may lead to the observed phenotype
- HCQ disturbs cell‐surface attachment of MSC‐derived and the extracellular matrix composition they produce
- Contrary to expected, SIM treatment also significantly decreased MSC‐derived osteoblast differentiation and mineralization

## Introduction

Hydroxychloroquine (HCQ) is an antimalarial agent now often used in systemic autoimmune diseases such as pSS, rheumatoid arthritis (RA) and systemic lupus erythematosus (SLE) due to its anti‐inflammatory properties 1, 2, 3. The pharmacokinetics of HCQ has been described extensively, but the exact mechanism of action remains unclear 4.

In addition to its anti‐inflammatory effects, the literature concerning the pharmacodynamics of HCQ is extensive. *In vivo* studies showed that HCQ has beneficial effects on the lipid profile of patients with RA and pSS by lowering serum levels of low‐density lipoprotein (LDL) cholesterol, triglycerides and total cholesterol as well as increasing high‐density lipoprotein (HDL) cholesterol 5, 6. Additionally, HCQ has been associated with beneficial cardiovascular and anticancer effects, but it is not used for these conditions as there are better alternatives available 7, 8.

*In vitro* studies have shown that HCQ is capable of inhibiting Toll‐like receptors (TLR) 7 and 9, which are involved in the pathogenesis of SLE 9, 10, 11. Although Raicevic *et al*. reported that osteoblasts do not express TLR 7 and 9, other studies did show TLR 9 expression in osteoblasts 12, 13, 14. HCQ has also been identified as an autophagy inhibitor by blocking the degradation of autophagosomes and promoting apoptosis in endometriosis, cervical cancer cells and myeloid leukaemia 15, 16, 17. In addition to the effects on autophagosomes, HCQ also acts on lysosomes. Some studies reported an increased lysosomal pH by HCQ treatment, which is associated with decreased lysosomal function 18, 19, while other studies did not observe a significant difference in lysosomal pH 10, 11. Furthermore, HCQ has been associated with increased lysosomal membrane permeabilization (LMP), a process occurring prior to mitochondrial membrane permeabilization (MMP) leading to apoptosis 20.

We recently reported that patients with pSS, of which the majority was using HCQ, had a higher BMD compared to healthy controls 21. Additionally, we found two studies showing a positive association between BMD and HCQ use in SLE patients, which was corrected for patient characteristics and disease activity 22, 23, while one study reported a negative effect of HCQ on BMD 24. We recently showed that HCQ leads to decreased osteoclast differentiation and activity due to HCQ treatment 25. Based on our previous studies, we have been suggested that HCQ stimulates the activity of the bone forming cells, the osteoblasts, which has not been studied before.

## Materials and methods

### Cell cultures

Human mesenchymal stromal cells (hMSCs; Lonza, Basel, Switzerland) were differentiated into osteoblasts as described before 26. Briefly, hMSCs were differentiated into mineralizing osteoblasts within 2–3 weeks, using dexamethasone and β‐glycerophosphate. The media were refreshed twice a week, and cells were treated without (control) and with HCQ (1 or 5 μg/ml). Alkaline phosphatase (ALP) activity was measured at day 7 of culture. Osteoblast mineralization was analysed by measuring the amount of precipitated calcium corrected for total protein at day 18 as extensively described before 26. Images were taken during culture to evaluate cell morphology. For microarray analysis, osteoblast cultures with and without 5 μg/ml HCQ were stopped at day 5.

### Mineralization staining assays

Calcium depositions were visualized with the Alizarin red staining assay as described before 26. Briefly, cells were fixed with 70% (vol/vol) ethanol and, after washing, stained for 10–20 min. with alizarin Red S solution. Phosphate depositions were visualized with the von Kossa staining assay as described before 26. Cells were washed with water, and the wells were stained for 30 min. with 5% silver nitrate (in bright daylight), incubated for 1 min. in 5% sodium carbonate in 25% formalin and finally for 2 min. in 5% sodium thiosulphate.

### Activation of simvastatin

hMSCs were differentiated to osteoblasts as described before. In addition, cells were treated with a dose range from 100 to 100 μM simvastatin (SIM; Sigma‐Aldrich, The Netherlands) with and without 5 μg/ml HCQ to evaluate whether the effects of HCQ on both osteoblast differentiation and mineralization could be antagonized by SIM. Simvastatin was activated before use as previously described 27. Briefly, 5 mg simvastatin was dissolved in 125 μl of 100% ethanol, with subsequent addition of 187.5 μl of 0.1 N NaOH. The solution was heated to 50°C for 2 hrs in a water bath and then activated by neutralizing to pH 7.0 using 0.1 N HCl. The resulting solution was brought to a final concentration of 4 mg/ml using distilled water, and aliquots were stored at 4°C until use.

### Immunocytochemistry assays

hMSCs were cultured for 5 days and stained for cytoskeletal actin. Briefly, cells were washed with phosphate buffer solution (PBS) and fixed with 10% formalin. PBS + Triton X‐100 was added for 10 min., followed by blocking aspecific binding sites, using PBS + Tween 0.05% + BSA 1% for 30 min. Cells were then incubated with a rhodamine‐conjugated phalloidin antibody for 1 hr at room temperature and washed with PBS + Tween 0.05% followed by DAPI staining. Staining of the cytoskeleton was visualized under a fluorescent microscope using a 535 nm filter. Additionally, a DAPI filter (365 nm) was used to visualize the nuclei and evaluate any apoptotic events (e.g. nuclear fragmentation, chromatin condensation).

For visualization and quantification of focal adhesions, cells were labelled for 1 hr with rabbit monoclonal anti‐vinculin antibody at 1:200 dilution at RT, followed by secondary Alexa Fluor 488 goat anti‐rabbit IgG at 1:400 dilution for a total of 1 hr 28.

### Illumina gene chip‐based gene expression

Total RNA of hMSCs was isolated as described before 26. Illumina Human HT‐12 v4 BeadChip (Illumina, Inc, San Diego, CA, USA) human whole‐genome expression arrays were used. RNA integrity of isolated RNA was assessed by RNA 6000 Nano assay on a 2100 Bioanalyzer (Agilent Technologies, Santa Clara, CA, USA). The RNA of 3 biologic replicates for each condition (control, 1 and 5 μg/ml HCQ) was analysed. The Illumina TotalPrep RNA Amplification Kit (Ambion, Austin, TX, USA) was used for RNA amplification of each sample according to manufacturer's instructions. In short, T7 oligo(dT) primer was used to generate single‐stranded cDNA, followed by a second‐strand synthesis to generate double‐stranded cDNA. *In vitro* transcription was performed to synthesize biotin‐labelled cRNA using T7 RNA polymerase. The cRNA was column purified and checked for quality by RNA 6000 Nano assay. A total of 750 ng of cRNA was hybridized for each array using the standard Illumina protocol, with streptavidin‐Cy3 (GE Healthcare, Piscataway, NJ, USA) being used for detection. Slides were scanned on an iScan and analysed using GenomeStudio (both from Illumina, Inc.).

### Microarray analysis

Background was subtracted from the raw data using GenomeStudioV2010.1 (Gene Expression Module 1.6.0, Illumina), and data were processed using the Bioconductor R3.3 lumipackage (www.bioconductor.org) 29. The data were transformed by variance stabilization and quantile normalization. Probes that were detected at least three times in the experiments (Illumina detection *P*‐value < 0.01) were considered to be expressed and were further analysed. Differentially expressed probes were identified using Bioconductor Package Limma (www.bioconductor.org), with adjusted *P*‐values adjusted to reduce the false discovery rate (FDR; *P* < 0.01) 30. Gene ontology (GO) analysis, selected Illumina IDs were analysed using the Database for Annotation, Visualization and Integrated Discovery (DAVID) 2008 hosted by the National Institute of Allergy and Infectious Diseases (NIAID) at the National Institutes of Health (Bethesda, MD, USA) and at GeneMANIA (http://www.genemania.org/). Merging of overlapping GO annotations was performed using the Reduce and Visualize Gene Ontology (REVIGO) tool (www.revigo.irb.hr) 31.

### Quantitative real‐time PCR analyses

The methods used for RNA extraction and cDNA synthesis and real‐time (RT)‐PCR have been described previously 26. Real‐time qPCR was performed using the ABI Prism 7900 sequence detection system (Applied Biosystems, Thermo Fisher Science, Bleiswijk, The Netherlands), and the results were analysed using SDS version 2.3 software (Applied Biosystems). Data are presented as relative mRNA levels calculated and corrected for gene expression of the housekeeping gene *GAPDH* by the formula: 2−Δ(Ct of gene of interest−Ct of housekeeping gene). All primers used are summarized in Table 1 **.**

**Table 1:** Primer sequences of the analysed genes

Gene	Forward primer	Reverse primer	
*GAPDH*	CCGCATCTTCTTTTGCGTCG	CCCAATACGACCAAATCCGTTG	
*TNC*	CACAGCCACGACAGAGGC	AAAGGCATTCTCCGATGCCA	
*ALP*	TAAAGCAGGTCTTGGGGTGC	GGGTCTTTCTCTTTCTCTGGCA	
*ACAT2*	GAGCTTTGCCTAGCTTGCAG	TGAAGGAACCTATGATGGTCCG	
*DHCR7*	GAGGTGTGCGCAGGACTTTA	CTTCTTGAACCGGCCCCTTA	
*CTSK*	TGCCCACACTTTGCTGCCGA	GCAGCAGAACCTTGAGCCCCC	
*CTNS*	AACGCGGTGCATTCCTGA	GCGTCTCCAAAGCAATCTGA	
*GPNMB*	TAAACCTTGAGTGCCTGCGT	TGAAATCGTTTGGCGGCATC	
*HMGCR*	TCTAGTGAGATCTGGAGGATCCAA	GGATGGGAGGCCACAAAGAG	

GO, gene ontology; *GAPDH*, Glyceraldehyde 3‐phosphate dehydrogenase; *TNC*, Tenascin C; *ALP*, Alkaline Phosphatase; *ACAT2*, Acetyl‐CoA Acetyltransferase 2; *DHCR7*, 7‐Dehydrocholesterol Reductase; *CTSK*, Cathepsin K; *CTNS*, Cystinosin, Lysosomal Cystine Transporter; *GPNMB*, Glycoprotein Nmb; *HMGCR*, 3‐hydroxy‐3‐methylglutaryl‐CoA reductase.

### Statistics

All results are expressed as means with standard error of the mean (S.E.M.) Comparisons of the continuous variables between three groups (control, 1 and 5 μg/ml HCQ) and two groups (control and 5 μg/ml HCQ) were performed using the one‐way analysis of variance (anova) and Student's *t*‐test, respectively. For anova analysis, the least significant difference post hoc test was used. A *P*‐value < 0.05 was considered significant. All analyses were performed in SPSS (version 21, IBM).

## Results

### HCQ inhibits osteoblast differentiation and activity

**Figure 1:**
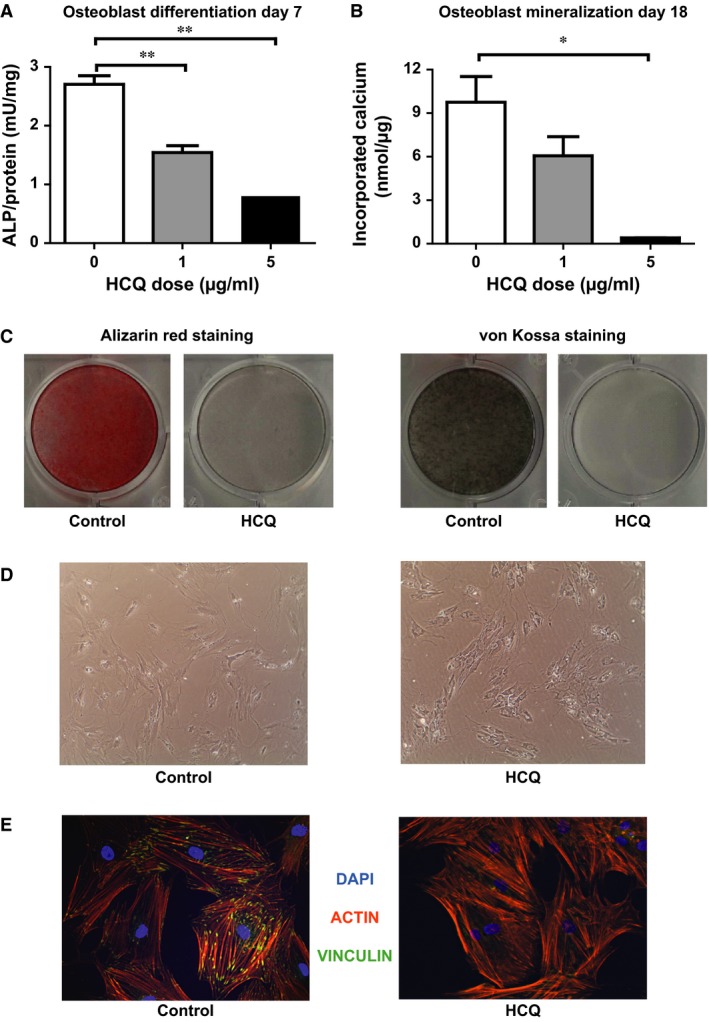
Effect of HCQ on MSC‐derived osteoblast differentiation and mineralization. All experiments are performed twice with N = 4 for every condition. (**A**) ALP measurement at day 7. (**B**) Mineralization at day 18 (**C**) Alizarin red staining and von Kossa staining in controls *versus* HCQ‐treated cells (**D**) Morphology of MSC‐derived osteoblasts at day 14 of culture in controls *versus* 5 μg/ml HCQ‐treated cells. (**E**) DAPI/Actin/Vinculin staining in controls *versus* 5 μg/ml HCQ‐treated cells. Data are presented as mean ± S.E.M. *P < 0.05, **P < 0.01. HCQ, hydroxychloroquine; ALP, alkaline phosphatase.

Osteoblast differentiation, as measured by ALP activity at day 7, was significantly decreased dose‐dependently between HCQ doses of 1 and 5 μg/ml *versus* controls (1.54 ± 0.11 mU/μg for HCQ dose 1 μg/ml and 0.8 ± 0.044 mU/μg for HCQ dose 5 μg/ml *versus* 2.7 ± 0.15 mU/μg for the controls, *P* < 0.001 for both and *P* < 0.001 for the dose‐dependent trend) (Fig. 1A). Mineralization at day 18 was significantly decreased between 5 μg/ml HCQ and controls (0.40 ± 0.015 nmol/μg *versus* 9.75 ± 1.76 nmol/μg, *P* = 0.011 and *P* < 0.001 for the dose‐dependent trend). In fact, using the highest HCQ dose, mineralization was virtually absent at 18 days of culture (Fig. 1B). Additionally, using alizarin red and von Kossa stainings, mineralization in the HCQ‐treated cells was absent compared to the controls (Fig. 1C). During culture, evaluation of the cells showed an altered morphology in the 5 μg/ml HCQ‐treated cells compared to the controls at day 14 (Fig. 1D). We performed vinculin stainings at day 5 of culture to analyse for differences in cell‐surface attachment between HCQ‐treated cells and controls. HCQ‐treated cells showed significantly less staining compared to the controls indicating less cell‐surface attachment due to HCQ (Fig. 1E). Furthermore, there is no evidence for a difference in apoptotic events between the conditions (data not shown) or cytoskeletal malformations (actin) between controls and HCQ‐treated cells based on rhodamine‐phalloidin staining (Fig. 1E).

### Microarray analysis of HCQ‐treated hMSCs yields 4 regulated processes in MSC‐derived osteoblasts

**Figure 2:**
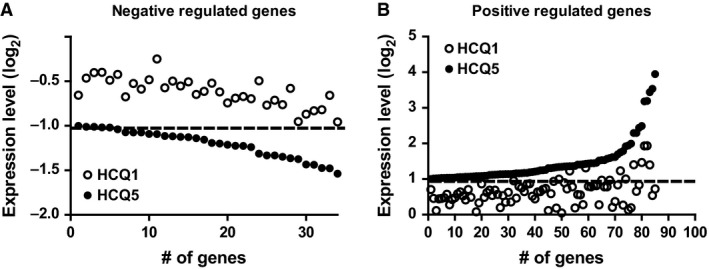
Dose–response curve of gene expression profiles between 1 and 5 μg/ml HCQ compared to controls. All experiments are performed with N = 4 for every condition. The dotted line indicates the threshold of twofold up‐ or down‐regulation. (**A**) Dose response for all genes that are negative regulated by HCQ. (**B**) Dose response for all genes that are positive regulated by HCQ. HCQ, hydroxychloroquine.

To gain insight into processes regulated by HCQ during osteoblast differentiation, we performed microarray gene expression analysis using Illumina Human HT‐12 v4 expression arrays. hMSCs were cultured and treated without or with HCQ (1 or 5 μg/ml) for 5 days as described above. Next, whole‐genome analysis of mRNAs was assessed following induction of osteogenic differentiation. When evaluating twofold up‐ and down‐regulated genes in HCQ‐treated cells *versus* controls, a clear dose response between 1 μg/ml and 5 μg/ml HCQ treatment was observed (Fig. 2A–B). In addition, none of the genes was stronger regulated by 1 μg/ml HCQ compared to 5 μg/ml HCQ. Therefore, we excluded the 1 μg/ml HCQ‐treated cells from further analysis. A total of 119 gene probes corresponding to 72 genes were differentially expressed (*q* < 0.05 and twofold change) at day 5 compared to controls. GO analysis of these gene probes resulted in a significant overrepresentation of 14 functional categories. Evaluation of the regulated genes within the categories showed a large overlap between the GO terms, and using REVIGO, we narrowed them down based on the largest number of genes to four main processes, namely (*i*) lipid metabolic process (GO:000629), (*ii*) developmental process (GO:0032502), (*iii*) lysosome (GO:0005764) and 4) extracellular matrix (GO:0031012) **(**Table 2 **)**.

**Table 2:** GO term enrichment analysis of 5 μg/ml HCQ treatment *versus* control at day 5 of MSC‐derived osteogenesis

GO	Name	Fold enrichment	Number of genes	*P*‐value
Biological process				
GO:0006629	Lipid metabolic process	4.1	20	0.0002
GO:0032502	Developmental process	2.0	37	0.014
Cellular component				
GO:0005764	Lysosome	9.9	11	0.0002
GO:0031012	Extracellular matrix	5.5	10	0.012

GO, gene ontology.

### PCR validation of HCQ‐regulated genes underlying selected GO terms from microarray analysis

**Figure 3:**
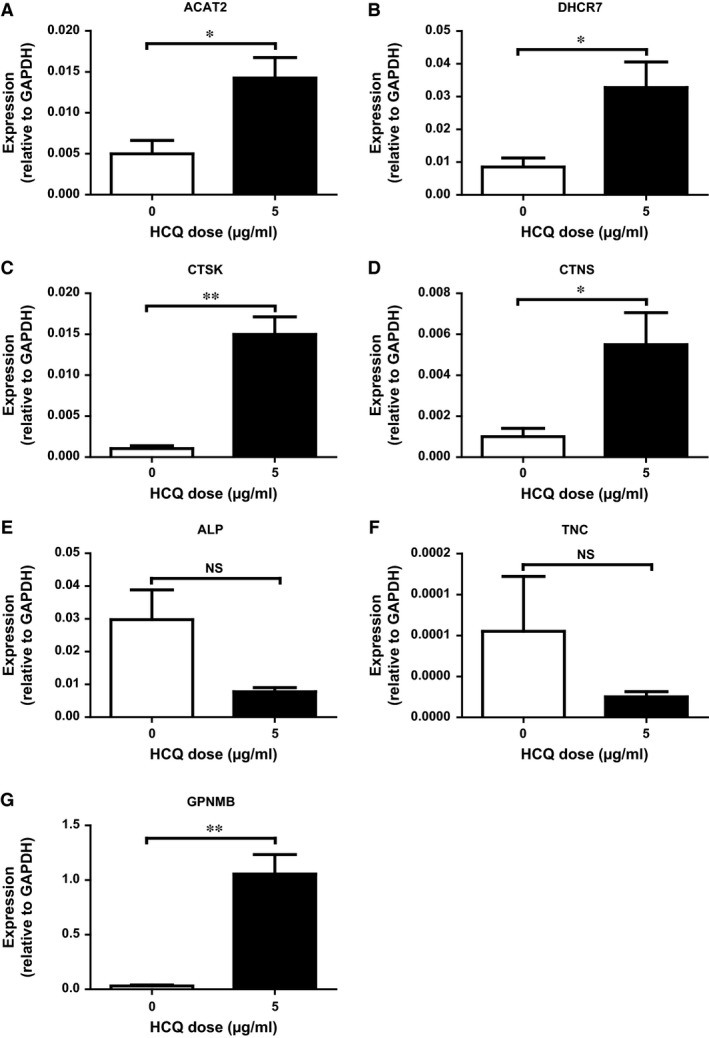
Validation of multiple genes regulated using real‐time qPCR. All experiments are performed with N = 4 for every condition. Total RNA was isolated from by 5 μg/ml HCQ‐treated hMSCs at day 5 followed by qPCR for (**A**) *ACAT2*, (**B**) *DHCR7*, (**C**) *CTSK*, (**D**) *CTNS*, (**E**) *ALP*, (**F**) *TNC* and (**G**) *GPNMB*. Gene expression was corrected for the housekeeping gene *GAPDH*. Data are presented as mean ± S.E.M. *P < 0.05, **P < 0.01. HCQ, hydroxychloroquine.

From every GO term, we selected two genes of interest for PCR validation (Table 2 **)**. For the GO term ‘lipid metabolism’ process, we selected acetyl‐CoA acetyltransferase 2 (*ACAT2*) and 7‐dehydrocholesterol reductase (*DHCR7*), which encode the first and last enzyme involved in the cholesterol biosynthesis pathway 32. For the GO term ‘extracellular matrix’, we selected tenascin C (*TNC*) and alkaline phosphatase (*ALP*) as these genes were highly regulated by HCQ and are known to be involved in osteoblast differentiation. Genes belonging to the GO term ‘lysosome’ include cathepsin K (*CTSK*) (a cysteine proteinase) and cystinosin, lysosomal cystine transporter (*CTNS*) (a small lysosomal membrane protein). All selected genes were also regulated in the GO term ‘developmental process’, and therefore, we only selected glycoprotein Nmb (*GPNMB*) from this GO term, as this was the strongest regulated gene upon HCQ treatment in our experiment. We validated these seven genes using real‐time PCR. Although expression of two genes (*ALP* and *TNC*) did not reach significance between controls and HCQ treatment, all genes showed the same direction of regulation compared to our results from the microarray analysis (Fig. 3A–G).

### Simvastatin decreases osteoblast differentiation and mineralization alone and in combination with 5 μg/ml HCQ

As HCQ up‐regulates the cholesterol synthesis pathway, we have been suggested that SIM (a cholesterol synthesis inhibitor) would antagonize the inhibitory effects of HCQ on osteoblast differentiation and mineralization. Therefore, we treated MSCs with SIM in multiple doses in the presence or absence of 5 μg/ml HCQ to evaluate the effects of SIM alone and in combination with HCQ on MSC‐derived osteoblasts. We found that SIM doses of 100 and 10 nM were ineffective, while SIM doses above 1 μM increased cell death in the early‐phase of the culture probably due to its cellular toxicity (data not shown).

**Figure 4:**
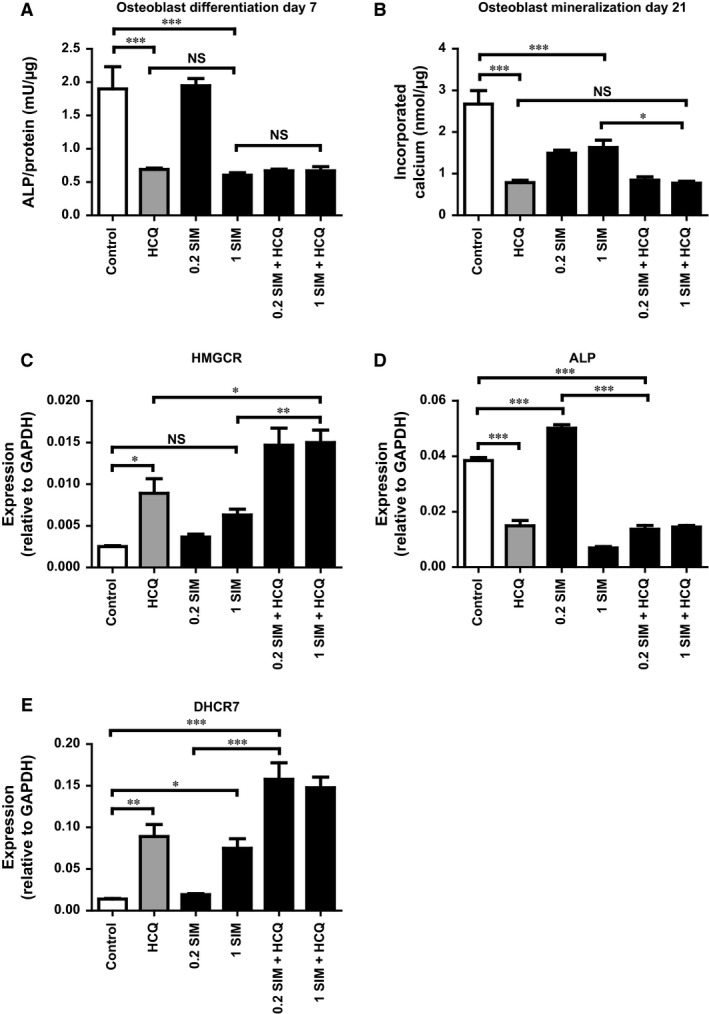
Effect of 5 μg/ml HCQ and SIM on MSC‐derived osteoblast differentiation and mineralization. All experiments are performed twice with N = 4 for every condition. SIM doses are 0.2 and 1 μM. (**A**) MSC‐derived osteoblast differentiation, as measured by ALP, at day 7 in HCQ‐ and/or SIM‐treated cells compared to control. (**B**) MSC‐derived osteoblast mineralization, as measured by calcium incorporation, at day 21 in HCQ and/or SIM‐treated cells compared to control. qPCR analysis of (**C**) *HMGCR,* (**D**) *ALP* and (**E**) *DHCR7* in HCQ‐ and/or SIM‐treated cells compared to control. Data are presented as mean ± S.E.M. *P < 0.05, **P < 0.01, ***P < 0.001. HCQ, hydroxychloroquine; SIM, simvastatin; ALP, alkaline phosphatase.

We showed that 1 μM SIM significantly decreased osteoblast differentiation, as measured by ALP activity, compared to untreated controls (0.67 ± 0.038 mU/μg for 1 μM SIM *versus* 1.9 ± 0.33 mU/μg for the controls, *P* < 0.001) (Fig. 4A). The effect of 1 μM SIM was similar to the effect of HCQ only as well as to the combination of these two drugs. Additionally, both 0.2 and 1 μM SIM significantly decreased osteoblast mineralization compared to the controls (1.49 ± 0.072 nmol/μg for 0.2 μM SIM and 1.63 ± 0.018 nmol/μg for 1 μM SIM *versus* 2.67 ± 0.32 nmol/μg for the controls, *P* < 0.001 for both) **(**Fig. 4B). However, the observed decreased mineralization by both doses of SIM was less severe compared to the HCQ treatment. The combination of HCQ with either SIM doses significantly decreased the mineralization compared to either SIM dose alone and is similar to the cells treated with HCQ only (1.63 ± 0.18 nmol/μg for 1 μM SIM *versus* 0.78 ± 0.59 nmol/μg for HCQ and 0.77 ± 0.047 nmol/μg for 1 μM SIM + HCQ, *P* < 0.05 for both).

We also analysed gene expression for *HMGCR* (the enzyme inhibited by SIM) in HCQ‐ and/or SIM‐treated cells compared to control. Although gene expression in SIM‐treated cells was higher, the effect was not significant. HCQ significantly increased *HMGCR* gene expression compared to controls (*P* < 0.05) (Fig. 4C). In addition, the combination with SIM and HCQ resulted in a significantly increased expression compared to either drug alone and to controls (*P* < 0.05 and *P* < 0.001, respectively). Furthermore, we analysed gene expression of *ALP* and *DHCR7* in HCQ‐ and/or SIM‐treated cells compared to control. Expression of *ALP* was significantly increased by 0.2 μM SIM compared to control (*P* < 0.001) (Fig. 4D). The combination of SIM and HCQ was similar to HCQ alone, but significantly lower compared to control (*P* < 0.001). Expression of *DHCR7* was significantly increased by both HCQ and 1 μM SIM compared to control (*P* < 0.01 and *P* < 0.05, respectively) (Fig. 4E). The combination of SIM and HCQ showed a synergistic effect leading to an increased *DHCR7* expression compared to control (*P* < 0.001) (Fig. 4E).

## Discussion

In the present study, we demonstrated that HCQ suppresses both MSC‐derived osteoblast differentiation and mineralization *in vitro*. Although some of the pharmacodynamics of HCQ may apply to specific biological processes in MSC‐derived osteoblasts, we did not come across studies reporting the direct effects of HCQ on MSC‐derived osteoblast differentiation or activity. Furthermore, we demonstrated results of the microarray analysis comparing 5 μg/ml HCQ‐treated hMSCs to controls. Up‐regulation of genes belonging to the cholesterol biosynthesis pathway, lysosomal pathway and extracellular matrix were the most significantly influenced processes by 5 μg/ml HCQ treatment. As SIM is a cholesterol synthesis inhibitor and beneficial for osteoblast differentiation and mineralization, we evaluated whether SIM could antagonize the negative effects of HCQ and enhance MSC‐derived osteoblast function simultaneously. Contrary to expected, SIM significantly decreased both MSC‐derived osteoblast differentiation and mineralization and the combination of SIM and HCQ yielded similar outcome compared to HCQ treatment alone.

As patients with pSS, of which the majority is using HCQ, have a higher BMD compared to healthy controls, we have been suggested that HCQ is beneficial for either MSC‐derived osteoblast differentiation or mineralization 21. However, our *in vitro* work showed that both MSC‐derived osteoblast differentiation (as measured by ALP activity) and mineralization (as measured by calcium incorporation and shown by mineralization stainings) are significantly decreased by 5 μg/ml HCQ treatment compared to controls.

We performed microarray analysis on both control and 5 μg/ml HCQ‐treated cells to assess potential mechanisms causing decreased MSC‐derived osteoblast differentiation and mineralization. We showed that the up‐regulation of genes involved in the cholesterol metabolism pathway was the most significantly regulated process by 5 μg/ml HCQ compared to control samples. From this pathway, 10 of 24 enzymes were significantly up‐regulated. Indeed, we confirmed the up‐regulation of this pathway by validating two of the involved genes (*ACAT2* and *DHCR7*) using RT‐PCR. Based on this finding, we speculate that either 1) HCQ has a direct positive regulatory effect on cholesterol synthesis or 2) HCQ causes an intracellular cholesterol depletion leading indirectly to increased cholesterol synthesis or increased cholesterol uptake. The latter is in agreement with the observed depletion of LDL cholesterol *in vivo* in patients that receive HCQ 5, 6.

The role of cholesterol in MSC‐derived osteoblast differentiation has mainly been studied by the use of statins (e.g. SIM). SIM inhibits 3‐hydroxy‐3‐methylglutaryl‐CoA reductase (HMGCR) and thereby blocks the synthesis of mevalonate and its downstream products leading to decreased levels of cholesterol 32. SIM activates Ras signalling by inhibiting the synthesis of cholesterol leading to overexpression of *BMP‐2* through the PI3K/Akt/MAPK pathway. BMP‐2 up‐regulates the expression of *RUNX2*, and phosphorylated RUNX2 stimulates a series of bone‐specific gene transcriptions and promotes the differentiation of osteoblasts 33, 34. Indeed, both *in vitro* and *in vivo* studies have reported the beneficial effects of statins on osteoblast differentiation and mineralization 35, 36. Additionally, *in vivo* studies showed that statins are a potential treatment for osteoporosis 37, 38. Based on these studies, we expected to find improved MSC‐derived osteoblast differentiation and/or mineralization and we speculated that SIM may antagonize the negative effects of HCQ. However, we found that both MSC‐derived osteoblast differentiation and mineralization were significantly decreased in SIM‐treated cells compared to controls. Furthermore, MSC‐derived osteoblast mineralization was significantly decreased by the combination of SIM and HCQ compared to cells treated with SIM only. A potential explanation might be that HCQ leads, due to an unknown mechanism, to an intracellular cholesterol depletion resulting in up‐regulation of cholesterol synthesizing enzymes as described earlier. Treatment with SIM would then block this compensatory mechanism of the cell which may lead to decreased MSC‐derived osteoblast development and activity. Indeed, gene expression of *HMGCR* is significantly increased in HCQ and SIM‐treated cells and it seems that both drugs have synergistic effects supporting our hypothesis. It remains unclear, however, why SIM did not have beneficial effects on MSC‐derived osteoblasts in our experiments. Another possible explanation might be the use of hMSCs, as many studies showing beneficial effects of SIM used different type of cell lines 39. A third explanation might be that 1 μM SIM has still toxic effects leading to impaired MSC‐derived osteoblast activity without leading to apoptosis. Despite using a dose–response experiment for SIM and following the methods as described in other papers, we could not confirm previously reported beneficial effects of SIM.

We showed a highly significant up‐regulation of the endosomal/lysosomal system by HCQ compared to the controls in our microarray analysis. Surprisingly, the most up‐regulated gene was *CTSK*, a lysosomal protease, which is predominantly known to be involved in bone resorption by osteoclasts 40, 41. The role of CTSK in osteoblasts is less well understood, and the majority of these studies are performed in mice. Mandelin *et al*. reported that osteoblast‐like cells indeed produce *CTSK* mRNA and release processed cathepsin K into culture media *in vitro* 42. A study performed in a *Ctsk* knockout mouse showed a significantly increased number of osteoblasts in the fracture callus with associated increased callus mineral density and strength compared to wild‐type mice 43. As we demonstrated a significantly decreased MSC‐derived osteoblast differentiation and mineralization and a significant up‐regulation of *CTSK* expression in HCQ‐treated MSC‐derived osteoblasts, a direct relation between *CTSK* up‐regulation and the observed phenotype is too premature at this stage.

According to literature, HCQ has been associated with increased LMP leading to apoptosis 20. LMP is caused by loss of cholesterol in the lysosomal membrane leading to the release of cathepsins and protons from the lysosomal lumen into the cytosol where they participate in apoptosis signalling 44. This may lead to the observed up‐regulation of *CTSK* gene expression in order to compensate for the loss. Additionally, cholesterol is identified as a stabilizer of the lysosomal membrane and may therefore counter LMP.

Finally, the decreased mineralization may be caused by HCQ‐induced alteration in the extracellular matrix (ECM) gene expression profile as this was one of the regulated GO terms following HCQ treatment. Eijken *et al*. reported that activin signalling in human osteoblasts changes the expression of a specific range of ECM proteins prior to the onset of mineralization, leading to a matrix composition with reduced or no mineralizing capacity 28. In agreement with this, we found similar ECM gene expression alterations due to HCQ treatment compared to controls in our microarray experiment (down‐regulation of *ALPL* and *CLEC3B*; up‐regulation of *POSTN*,* MMP7* and *MMP15*). In addition, we showed that staining for yet another ECM protein, vinculin, was significantly decreased in HCQ‐treated cells compared to controls. Therefore, we speculate that HCQ leads to reduced cell‐surface attachment and altered ECM composition leading to decreased matrix mineralization.

Based on these findings, our final hypothesis is that HCQ ‘attacks’ the lysosomal membrane by removing cholesterol leading to decreased osteoblast differentiation and mineralization. As a compensatory mechanism, both the cholesterol synthesis pathway and the lysosomal pathway are up‐regulated in an attempt to restore osteoblast function. In addition, HCQ may also affect ECM composition leading to decreased cell attachment, differentiation and matrix mineralization. The discrepancy between high BMD and decreased MSC‐derived osteoblast function due to HCQ treatment might be caused by systemic factors that stimulate bone formation and/or systemic or local factors that reduce bone resorption which is lacking in cell cultures. In fact, we have shown that HCQ strongly suppresses bone resorption *in vitro* and *in vivo* and in women with a high bone turnover state, this may lead to a net increase in bone mass 25.

The strength of this study is that we performed an unbiased evaluation of potential mechanisms of action for the observed decreased MSC‐derived osteoblast differentiation and mineralization using microarrays. Additionally, genetic data from the microarray were translated into functional experiments, but the precise mechanism remains elusive.

In conclusion, we demonstrated that HCQ suppresses MSC‐derived osteoblast differentiation and mineralization *in vitro*. Furthermore, we reported results of our microarray analysis showing significant up‐regulation of the cholesterol biosynthesis and lysosomal pathway. Surprisingly, treatment with SIM and HCQ also resulted in decreased MSC‐derived osteoblast differentiation and mineralization. A potential mechanism could be HCQ‐induced LMP leading to decreased MSC‐derived osteoblast development and activity.

## Conflict of interest

All authors declare that they do not have Conflict of interest.
